# Translational strategy using multiple nuclear imaging biomarkers to evaluate target engagement and early therapeutic efficacy of SAR439859, a novel selective estrogen receptor degrader

**DOI:** 10.1186/s13550-020-00646-w

**Published:** 2020-06-29

**Authors:** Laurent Besret, Sébastien d’Heilly, Cathy Aubert, Guillaume Bluet, Florence Gruss-Leleu, Françoise Le-Gall, Anne Caron, Laurent Andrieu, Sylvie Vincent, Maysoun Shomali, Monsif Bouaboula, Carole Voland, Jeffrey Ming, Sébastien Roy, Srinivas Rao, Chantal Carrez, Erwan Jouannot

**Affiliations:** 1 Sanofi Research and Development France, 13 quai Jules Guesde, 94403 Vitry-sur-Seine, France; 2 Present address: Takeda Pharmaceuticals, 35 Landsdowne St, Cambridge, MA 02139 USA; 3 Sanofi Research and Development USA, 640 Memorial Drive, Cambridge, MA 02139 USA; 4 Sanofi Research and Development France, 371, rue du Pr Blayac, 34184 Montpellier Cedex 4, France; 5 Sanofi Research and Development USA, 55 Corporate Drive, Bridgewater, NJ 08807 USA

**Keywords:** SAR439859, Estrogen-positive breast cancer, SERD, Target engagement, Combination therapy, Palbociclib, Positron emission tomography, [^18^F]-FDG, [^18^F]-FES, [^18^F]-FLT

## Abstract

**Purpose:**

Preclinical in vivo nuclear imaging of mice offers an enabling perspective to evaluate drug efficacy at optimal dose and schedule. In this study, we interrogated sufficient estrogen receptor occupancy and degradation for the selective estrogen receptor degrader (SERD) compound SAR439859 using molecular imaging and histological techniques.

**Material and methods:**

[^18^F]FluoroEstradiol positron emission tomography (FES-PET), [^18^F]FluoroDeoxyGlucose (FDG) PET, and [^18^F]FluoroThymidine (FLT) PET were investigated as early pharmacodynamic, tumor metabolism, and tumor proliferation imaging biomarkers, respectively, in mice bearing subcutaneous MCF7-Y537S mutant ERα+ breast cancer model treated with the SERD agent SAR439859. ER expression and proliferation index Ki-67 were assessed by immunohistochemistry (IHC). The combination of palbociclib CDK 4/6 inhibitor with SAR439859 was tested for its potential synergistic effect on anti-tumor activity.

**Results:**

After repeated SAR439859 oral administration over 4 days, FES tumoral uptake (SUVmean) decreases compared to baseline by 35, 57, and 55% for the 25 mg/kg qd, 12.5 mg/kg bid and 5 mg/kg bid treatment groups, respectively. FES tumor uptake following SAR439859 treatment at different doses correlates with immunohistochemical scoring for ERα expression. No significant difference in FDG uptake is observed after SAR439859 treatments over 3 days. FLT accumulation in tumor is significantly decreased when palbociclib is combined to SAR439859 (− 64%) but not different from the group dosed with palbociclib alone (− 46%). The impact on proliferation is corroborated by Ki-67 IHC data for both groups of treatment.

**Conclusions:**

In our preclinical studies, dose-dependent inhibition of FES tumoral uptake confirmed target engagement of SAR439859 to ERα. FES-PET thus appears as a relevant imaging biomarker for measuring non-invasively the impact of SAR439859 on tumor estrogen receptor occupancy. This study further validates the use of FLT-PET to directly visualize the anti-proliferative tumor effect of the palbociclib CDK 4/6 inhibitor alone and in combination with SAR439859.

## Introduction

Both endogenous and exogenous steroid hormones such as estrogen and progesterone have been implicated in the pathogenesis of breast cancer. Clinical treatment decisions are driven by the expression of estrogen receptor (ER), progesterone receptor (PR), and human epidermal growth factor receptor-2 (HER2) and the classification of breast tumors according to their receptor status into HER2-positive, ER-positive/HER2-negative, and triple-negative clinical subtypes. About 70–75% of breast cancers express ERα which is a hormone regulator transcription factor [1]. ERα-positive breast cancers respond well to therapy targeting ERα signaling either through competitive binding of ER antagonists such as tamoxifen or by blocking the production of estrogen by aromatase inhibitors [2]. However, despite the benefits of those therapies, de novo and acquired resistance remain an issue in many patients [3].

Several strategies have been used in the clinic to further target the ER pathway including the development of oral selective estrogen receptor downregulators/degraders (SERDs). Given the efficacy of fulvestrant, the first-in-class intramuscular SERD compound, oral SERDs are likely to play an important role in therapeutic management of ER+ tumors. This class of antagonists induces a degradation of the ERα receptor; their potential to block endocrine- and non-endocrine-dependent signaling has been recognized to offer a therapeutic approach in early-stage disease and improves outcomes for those with advanced ER+-resistant breast cancer [4, 5]. Fulvestrant exhibits both poor physicochemical and pharmacokinetic properties and the intramuscular route of administration has also limited its use. A new generation of SERDs with improved properties is being currently developed [6–8].

SAR439859 is an oral ER antagonist and selective ER degrader (SERD) with potent anti-tumor activity regardless of ESR1 mutation status, and this compound is proposed for the treatment of locally advanced or metastatic ER-positive breast cancer. SAR439859 binds with high affinity to human wild-type ERα as well as to mutants ERα (ERα-Y537S and ERα-D538G). SAR439859 not only antagonizes the binding of estradiol to ER but also promotes the transition of ERα to an inactive conformation that leads to as up to 98% receptor degradation at nanomolar concentrations in cellular assays. These dual properties of SAR439859 translate in a robust inhibition of ERα pathways and a more effective anti-proliferative activity in ERα-dependent breast cancer cell lines driven by mutant or wild-type ERα compared to other ERα inhibitors [9]. SAR439859 induces ERα degradation and inhibits in a dose-dependent manner the cell proliferation of MCF7 cells harboring wild-type ERα or mutant ERα. Cell growth inhibition was also shown in a larger panel of ERα-positive breast cancer cell lines. SAR439859 has no effect on the growth of ERα-negative cell line confirming the selective effect of SAR439859 to ERα. In nude mice bearing the ERα-positive MCF-Y537S tumor model, a single oral administration of SAR439859 over 2.5–25 mg/kg dose range induced a dose-dependent intra-tumoral degradation of ERα. At 12.5 mg/kg, SAR439859 decreased ERα by 90% for at least 8 h [9]. In vivo efficacy data obtained against MCF7-Y537S demonstrated that the tumor growth inhibition induced by SAR439859 was correlated with ERα intra-tumoral degradation. Of note SAR439859 induced better tumor regression at 12.5 mg/kg twice a day regimen when compared to the 25 mg/kg daily regimen over 3 weeks (internal data). Since SAR439859 administration at 12.5 mg/kg was shown to maintain ERα decrease for 8 h, this suggests that continuous target occupation/inhibition is required to achieve tumor regression.

Positron emission tomography (PET) is described as the most valuable technique to measure non-invasively the target engagement in preclinical and clinical settings [10]. Using [^18^F]-FluoroEstradiol (FES) as a PET tracer enables evaluation of ER expression/function in patients with breast cancer. FES-PET may thus emerge as a valuable tool to predict which patients with primary, recurrent, or metastatic breast cancer will respond to hormone therapy [11]. Quantification of target engagement is a prerequisite for receptor occupancy studies and would provide a means to relate dosage of drug to the occupancy of target receptor by the drug candidate. A good correlation exists between [^18^F]-FES uptake and ERα expression measured by immunohistochemistry (IHC) [12]. However, the question remains whether FES-PET can predict the response to anti-estrogen therapy in patients. In a study published in 2001, Mortimer et al. [13] concluded that the level of [^18^F]-FES uptake at baseline predicts the response to tamoxifen treatment. Linden et al. [14] confirmed that the baseline measurement of [^18^F]-FES uptake (SUV < 1.5–2.0) would predict responses to targeted hormonal therapy. More recently, Chae et al. [15] in a neoadjuvant therapy showed that ER-rich patients with low [^18^F]-FES uptake (SUVmax < 7.3) are more susceptible to better respond to neoadjuvant chemotherapy compared to neoadjuvant endocrine therapy. In a recent review, van Kruchten [16] described the potential of FES-PET imaging in providing information about ER binding of endocrine drugs. Gong et al. [17] conducted a preliminary study to monitor the change in [^18^F]-FES uptake post-therapy and concluded that [^18^F]-FES could be a predictive indicator in patients receiving docetaxel combined or not with fulvestrant. Of note, to date only a few clinical trials support FES-PET imaging as a tool for selecting the therapeutic dose of selective ERα inhibitors [18, 19]. Clinical investigations with SAR439859 are currently under way; FES-PET imaging was retained to select the dose in a cohort of breast cancer patients (clinical trial #NCT03284957). In this respect, FES-PET as direct imaging technique can provide robust and valuable datasets for assessment of ERα modulation by SAR439859.

Recently, Christofanilli et al. [4] published data of a clinical trial aiming at comparing the effect of fulvestrant combined to a cyclin-dependent kinase (CDK) 4/6 inhibitor (palbociclib) versus fulvestrant plus placebo; patients treated with the combined regimen had a significant improvement in progression-free survival. Numerous clinical trials in cancer breast patients are currently being performed with CDK 4/6 inhibitors (for review see [20]); their favorable characteristics in terms of toxicity and route of administration make them of great interest for combination with endocrine therapy [21]. In nude mice bearing MCF7-Y537S tumor model, combining SAR439859 with palbociclib led to further growth-inhibitory effects compared with monotherapy alone [22] suggesting that the combination of SAR439859 with palbociclib could bring therapeutic benefit to ERα-positive breast cancer patients.

The preclinical work reported herein presents the use of FES-PET for monitoring response to SAR439859 endocrine therapy in SCID mice bearing a mouse xenograft model of ERα+ breast cancer. We assessed whether the target occupancy measured with FES-PET imaging could help select the best regimen to maximize the drug’s efficacy. To assess the feasibility of measuring therapeutic efficacy early in the course of treatment with SAR439859 and palbociclib, tumor metabolism and tumor proliferation were also investigated using [^18^F]FluoroDeoxyGlucose (FDG) PET and [^18^F]FluoroThymidine (FLT) PET.

## Material and methods

### PET radiotracers and imaging procedure

[^18^F]-FES production was established in house. The process was settled-up on a TRASIS All-In-One® automated synthesizer. The radio-synthesis of [^18^F]-FES (3) was performed in three steps starting from commercial 17b-epiestriol-O-cyclic sulfone (MMSE, ABX GmbH) (1) as follows: (a) nucleophilic substitution from [^18^F]-KF, (b) hydrolysis of the protected intermediate (2) in acidic media, and (c) semi-preparative HPLC purification.

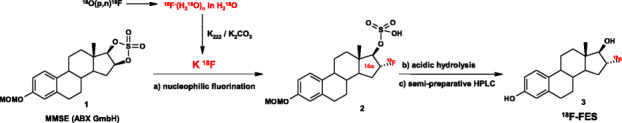



Aqueous cyclotron-produced [^18^F]-fluoride was absorbed onto QMA anionic resin cartridge (from ABX) and then released with a solution of K_2_CO_3_ (1.12 mg; 8.1 μmol) and K222 (6 mg, 15.9 μmol) in MeCN/H_2_O (83:17 v/v; 900 μL). The resulting mixture was recovered into a reactor and dried by heating under a nitrogen stream. MMSE 1 (0.9 mg, 2.4 μmol) in DMSO (1.2 mL) was introduced into the reactor. The reactor was sealed using the built-in pinch valves then stirred for 6 min at 105 °C. Aqueous H_2_SO_4_ (1 M, 1 mL) was added. The reactor was closed, and the reaction mixture was stirred for 6 min at 100 °C. After cooling to 40 °C, the crude mixture was diluted in H_2_O (4 mL) and was injected onto a semi-preparative HPLC module equipped with a semi-preparative column (Kromasil C18, 250 × 10mm, 5 μm, elution H_2_O/EtOH 40/60 at 3 ml/min), a UV detector fixed at *λ* = 254 nm and a radiation detector (Flumo, Berthold). [^18^F]-FES (*n* = 21; RCY up to 40% decay corrected) was collected in a vented sterile vial through a 0.22-μm filter and formulation was performed with saline.

Medical-grade [^18^F]-FLT (1000 MBq/mL) and [^18^F]-FDG (185 MBq/mL) were purchased from PETNET Solutions SAS (France) and IBA Molecular SA (France), respectively.

PET/CT was performed using a preclinical INVEON PET/CT system (Siemens Medical Solutions USA, Inc.). For imaging, the mice were injected intravenously with the selected radiotracer and kept conscious during tracer uptake in a heated box. Mice were isoflurane-anesthetized by trained personnel during the scans, and body temperature was maintained at 37 °C. FES-PET and FDG-PET scans were performed 60 min after injection of the radiotracer. FLT-PET scans were acquired 90 min post-tracer injection. CT acquisition (500 μA; 80 kVp) time duration was 5 min followed by 10 min for PET imaging (level energy thresholds, 350–650 KeV). Images were reconstructed using a two-dimensional ordered subset-expectation maximization reconstruction algorithm (OSEM2D). Image analysis was performed using Inveon Research Workplace 4.2 software (Siemens Medical Solutions USA, Inc.); 3D regions of interest for the tumor were defined on the CT image and transferred to the co-registered PET image. Standardized uptake value (SUV) in tumor is *C*(*t*)/ID × BW where *C*(*t*) is the radioactivity activity concentration [kBq/ml] measured by the PET scanner within a region of interest, ID is the decay-corrected amount of injected radiolabeled tracer [kBq], and BW is the weight of the mouse [g]. SUV was calculated for each mouse in each experimental group. Mean SUV (SUVmean) ± SD values were calculated for each experimental group.

### Tumor implantation and subsequent examination

Animals were maintained according to the institutional guidelines and approval by local authorities. CB17/lcr-Prkdcscid/crl (severe combined immunodeficiency—SCID) mice, at 6–8 weeks old, were bred at Charles River France (Domaine des Oncins, 69210 L’Arbresle, France) from strains obtained from Charles River, USA. Mice were over 17 g at the start of the study after an acclimatization time of at least 5 days. They had free access to food (UAR reference 113, Villemoisson, 91160 Epinay sur Orge, France) and water. They were housed on a 12-h light/dark cycle. Environmental conditions including animal maintenance, room temperature (22 °C ± 2 °C), relative humidity (55% ± 15%), and lighting times were recorded.

The MCF7 human caucasian breast adenocarcinoma was purchased at the American Type Culture Collection (ATCC® HTB-22™, Rockville, MD, USA). This tumor cell line was engineered in-house to express the activated mutant form Y537S of the estrogen receptor. The MCF7-Y537S breast cancer model was established by implanting subcutaneously 2.10^7^ tumor cells in SCID female mice which were not supplemented in estradiol as the mutation of ESR1 (Y537S) leads to constitutive activation of ERα [23]. The model was maintained by subcutaneous serial passages in female SCID mice once every 3–4 weeks. For the experiments, 8-week-old female SCID mice were subcutaneously implanted (on day 0) with fragments of tumor.

Tumor volume (length × width^2^/2) was monitored biweekly. When tumor load reaches the required size, animals were randomized on tumor size or on radiotracer uptake after baseline imaging when relevant. Animals were treated with vehicle or compound and imaged according to the protocol. After the final imaging session, animals were sacrificed; tumors were excised and then processed for immunohistochemistry (IHC).

### Study design—targeted molecular therapy and PET/CT examination

#### Target engagement study measured with [^18^F]-FES after a single administration of SAR439859 (experiment #1)

A dose-escalation study with SAR439859 was performed to assess dose-dependency in [^18^F]-FES tracer tumor uptake. Three dose groups (1.25, 5, and 12.5 mg/kg) were compared to a group receiving vehicle (*n* = 3–5 mice per group); the 3 treatment groups received a single administration per oral route of the compound on day 27 post-tumor implantation for a tumor load of 170–350 mg. Dose range was chosen considering in vivo anti-tumor activity observed at 12.5 mg/kg bi-daily (bid) with caliper measurements. FES-PET imaging was performed 4 h post-treatment, a time at which the maximum of ERα degradation was previously documented by western blot measures [9].

#### Target engagement measured with [^18^F]-FES for different SAR439859 drug regimen (experiment #2)

[^18^F]-FES uptake was assessed prior to the drug injection for each mouse (baseline at time T0 corresponding to day 22 for a tumor load of 180–288 mg). Based on the [^18^F]-FES uptake at baseline, animals were randomized into 3 treatment groups (*n* = 9 mice per group) and 1 vehicle group (*n* = 9). Treatment with SAR439859 was orally administrated at 25 (daily qd), 12.5 (bid), and 5 (bid) mg/kg under 10 mg/mL for 4 days starting on day 26 until day 29. The mice in the vehicle group were injected with 10 mL/kg vehicle (bid). Post-treatment FES-PET imaging was performed on day 29. Mice were then sacrificed, and the tumors were removed for ERα-IHC analysis.

#### Therapeutic efficacy measured with [^18^F]-FDG imaging (experiment #3)

When the MCF7-Y537S tumor burden reached the desired range (144–600 mg), animals were randomized into 3 treatment groups (*n* = 9 mice per group) and 1 vehicle group (*n* = 9). The mice in the treatment groups were orally administered over 3 days as follows (from day 34 to day 37): SAR439859 alone (5 mg/kg/adm bid), palbociclib alone (100 mg/kg/adm qd), and the combination of SAR439859 and palbociclib under the same regimen. The mice in the vehicle group were injected with vehicle (bid). FDG-PET imaging was performed at baseline and under treatment at 18 h (day 35) and 42 h (day 36) post-first administration (day 34). In addition, FES-PET imaging was performed on the last day (37) of the study. Mice were then sacrificed, and the tumors were removed for Ki-67 IHC analysis.

#### Therapeutic efficacy measured with [^18^F]-FLT imaging (experiment #4)

Animals bearing MCF7-Y537S xenografts were imaged with [^18^F]-FLT at baseline on day 18 (tumor load of 63–256 mg). Subsequently, based on FLT-PET signal at baseline the animals were distributed into a vehicle-treated group and four treatment groups. The mice were treated for 4 days (from day 21 to day 25) via oral route with SAR439859 (5 mg/kg/adm; bid; *n* = 8), SAR439859 (12.5 mg/kg/adm; bid; *n* = 7), palbociclib (100 mg/kg/adm; qd; *n* = 8), and the combination of SAR439859 (5 mg/kg/adm; bid) with palbociclib (100 mg/kg/adm; qd; *n* = 8). Following the treatment, FLT-PET imaging was performed 72 h post-first treatment (day 24). FES-PET was also carried out on day 25 in order to assess the level of ERα engagement.

The objectives of each experiment have been summarized in Table 1.

**Table 1:** Details and objectives of the experiments conducted

Study	Imaging biomarker	Treatment	Objectives
Experiment #1: target engagement after SAR439859 single injection	FES-PET 4 h post-therapy	Single SAR439859 administration; doses tested: 1.25, 5, 12.5 mg/kg	Measure dose effect on [^18^F]-FES tumor uptake after a single administration of SAR439859 over 1.25–12.5 mg/kg range of doses
Experiment #2: target engagement after SAR439859 repeated injections	FES-PET (baseline vs post-therapy)	4 days SAR439859 treatment: - 5 mg/kg, bid - 12.5 mg/kg, bid - 25 mg/kg, qd	Measure target engagement after repeated SAR439859 treatments over 5–25 mg/kg range of doses, correlate target engagement with IHC, compare dose regimens
Experiment #3: tumor metabolism evaluation under SAR439859 treatment	FDG-PET (baseline vs under treatment at 18 h and 42 h after start of therapy) FES-PET (post-therapy to confirm target engagement)	3 days treatment: - SAR439859 5 mg/kg, bid - Palbociclib 100 mg/kg, qd - Combination	Measure tumor metabolism under SAR439859 as single agent or in combination with palbociclib
Experiment #4: tumor proliferation evaluation post SAR439859 treatment	FLT-PET (baseline vs post-therapy) FES-PET (post-therapy to confirm target engagement)	4 days treatment: - SAR439859 5 mg/kg, bid - Palbociclib 100 mg/kg, qd - Combination	Measure tumor proliferation after 4 days of SAR439859 treatment as single agent or in combination with palbociclib

### Immunohistochemistry

For experiments #2, #3, and #4, terminally sampled tumors were fixed in 4% formalin at 4 °C and then transferred in 10% neutral buffered formalin at 4 °C for 5 days. The formalin-fixed and paraffin-embedded blocks (FFPE) were sectioned at 5-μm thickness on a microtome; adjacent tissue slices for the expression of markers were transferred onto Superfrost Plus glass slides for immunohistochemistry studies.

FFPE sections were performed using Ventana Discovery XT automated System (Ventana Medical Systems, Inc., USA) on de-waxed and rehydrated slides. For all immunostaining, antigen retrieval procedure was applied with cell conditioning 1 (CC1) buffer (Tris/Borate/EDTA, pH 8) standard condition. Following endogenous avidin and biotin blocking treatment (760-050, Ventana), sections were incubated with primary antibody (ready to use for anti-ER, clone SP1 ref 790-4324-ROCHE or anti-huKi67 Mab mouse IgG1, clone MIB-1 ref M7240-DAKO antibody) for 1 h in PBS at room temperature. A post-fixation step with glutaraldehyde (0.05% in NaCl 0.9% w/v) for 4 min at room temperature was done. The secondary antibody goat anti-rabbit (1/200) biotin-SP-AffiniPure IgG, or goat anti-mouse conjugated biotin IgG (at final concentration of 2.5 μg/ml diluted in buffer (760-108, Ventana)) was incubated at room temperature for 32 min.

All immunostaining was done with DABMap™ chromogenic detection kit according to the manufacturer’s recommendations. Immunostained sections were subsequently counterstained (790-2208, Ventana) and bluing reagent was applied (760-2037, Ventana Medical Systems, Inc., USA). Stained slides were dehydrated, and cover slipped with Clearvue Moutant XYL ref 4212-Thermoscientific. Slides are digitized using Aperio AT2—Leica. Positive signals are quantified by image analysis system (Scanscope) allowing identifying staining intensity (from 1 for “light signal,” 2 for “medium signal,” and 3 for “strong signal”) and the percentage of nuclei at intensity 1, 2, and 3 were recorded. The results are reported as H-score which summated the percentage of area stained at each intensity level multiplied by the weighted intensity [24]: H-score = 1 × (% nuclei at intensity 1) + 2 × (% nuclei at intensity 2) + 3 × (% nuclei at intensity 3).

### Statistical analysis

#### Experiments 1 and 2

Pairwise two-sided Wilcoxon tests versus vehicle corrected with a Bonferroni-Holm adjustment were performed on the change from baseline of [^18^F]-FES-PET (SUV) parameter in order to compare each dose of SAR439859 to the vehicle group. For experiment 2, a Pearson coefficient of correlation was calculated between FES-PET (SUV) and the IHC H-score parameter, whatever the treatment.

#### Experiment 3

For FDG-PET imaging, a two-way analysis of covariance (ANCOVA) was performed on SUV change from baseline, with group, day, and group by day interaction as fixed effect and the baseline as covariate. Pairwise comparisons of each treatment were performed for each day. Tukey-Kramer adjustment was then used for multiplicity issues of test within each day. Estimates of differences with associated 95% confidence intervals (CI) are also provided.

For the other parameters, a one-way analysis of variance (ANOVA) was performed on raw data, with group as fixed effect. Pairwise comparisons of each treatment were performed using a Tukey-Kramer adjustment for multiplicity issues. Estimates of differences with associated 95% confidence intervals (CI) are also provided.

#### Experiment 4

For FLT-PET imaging, a one-way analysis of covariance (ANCOVA) was performed on SUV with group as fixed effect and the baseline as covariate. All pairwise comparisons of treatment groups were performed with Tukey-Kramer adjustment for multiplicity.

## Results

### Target engagement study measured with [^18^F]-FES after a single administration of SAR439859 (experiment #1)

**Fig. 1:**
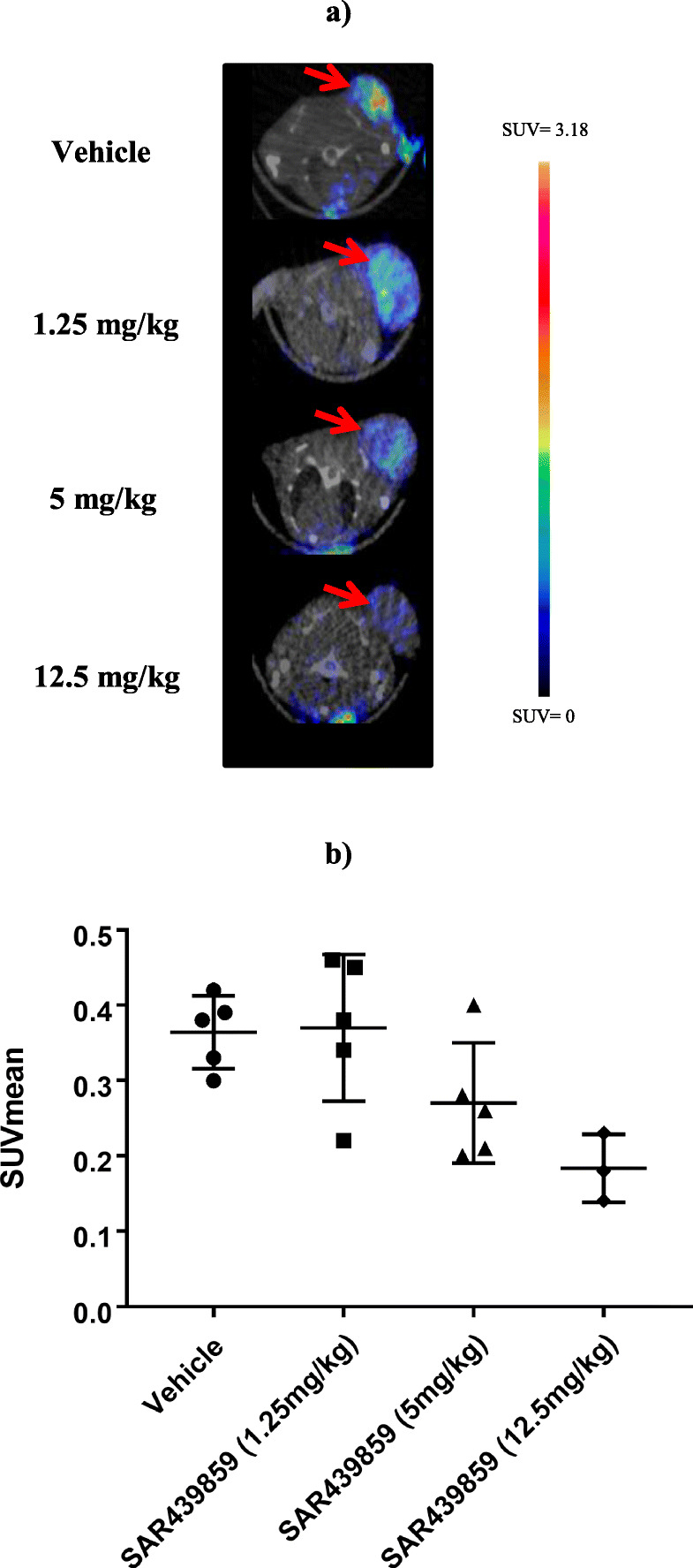
**a** Representative co-registered axial FES-PET/CT images in vehicle- or SAR439859-treated mice (the red arrow indicates tumor). **b** Quantitative analysis of FES-PET signal showing a dose-dependent reduction 4 h after a single administration of SAR439859; the data are expressed as SUVmean (mean ± SD)

Our primary aim in preclinical studies was to assess *in vivo* the quantitative changes of SAR439859-induced ER occupation/degradation using non-invasive imaging. Based on ERα degradation kinetic profile (internal *in vitro* data), subjects receiving a single dose of SAR439859 were scanned 4 h after dosing (Tmax for FES-PET imaging at tumor SAR439859 Cmax) to determine the pharmacodynamic relationship between SAR439859 dose and ERα occupancy in MCF7-Y537S tumor-bearing mice. As shown in Fig. 1, [^18^F]-FES SUV levels measured 4 h post-therapy decreased in a dose-dependent manner (1.25 mg/kg: SUVmean = 0.37 ± 0.10; 5 mg/kg: SUVmean = 0.27 ± 0.08; 12.5 mg/kg: SUVmean = 0.18 ± 0.04 vs control: SUVmean = 0.37 ± 0.05) reflecting increasing ERα occupancy with increasing doses. [^18^F]-FES uptake reduction of 50% was measured in the 12.5 mg/kg group. Although there was no statistically significant difference between groups, likely related to the limited number of observations, the results support a trend for a dose-response effect of SAR439859.

### Target engagement measured with [^18^F]-FES for different SAR439859 drug regimens (experiment #2)

**Fig. 2:**
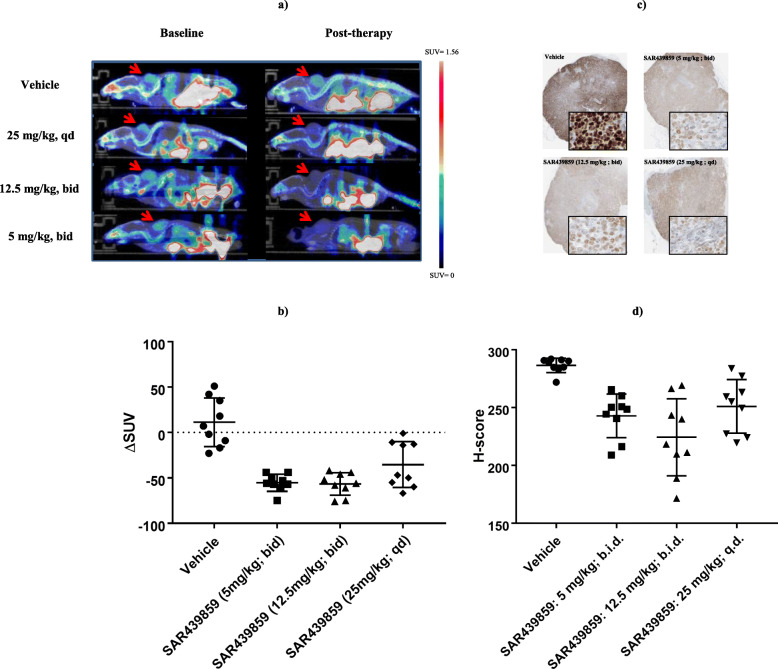
**a** Co-registered FES-PET/CT imaging of MCF7-Y537S xenograft tumor-bearing mice at 8 h (bid regimen) or 24 h (qd regimen) after 4 days of administration of SAR439859. **b** Individual data and mean ± SD of ΔSUVmean for [^18^F]-FES uptake during different regimen of SAR439859 treatment. **c** ERα staining and **d** ER-positive cells quantification in vehicle and SAR439859-treated tumor samples

As a second step, our goal was to determine if different SAR439859 drug regimens modulate tumor [^18^F]-FES uptake. The [^18^F]-FES SUV was measured in each mouse when tumor load was in the median range 180–288 mg on day 22; the SUV was in the range 0.216 to 0.995 in 36 scanned animals (Fig. 2). Vehicle and treatment groups were constituted in order to get a homogeneous SUV distribution for baseline uptake. Day 29 post-treatment [^18^F]-FES uptake was found to be stable as compared to baseline in the vehicle group (0.43 ± 0.07 vs 0.40 ± 0.12) and decreased in all the treatment groups: 0.24 ± 0.06 vs 0.41 ± 0.12 at 25 mg/kg qd, 0.17 ± 0.07 vs 0.41 ± 0.12 at 12.5 mg/kg bid, and 0.20 ± 0.04 vs 0.48 ± 0.22 at 5 mg/kg qd. The tumor uptake of [^18^F]-FES in the treatment groups decreased significantly for all dosages tested compared to vehicle (*p* < 0.02). Compared to their baseline values, SUVmean values decreased by 35, 57, and 55% for the 25 mg/kg qd, 12.5 mg/kg bid, and 5 mg/kg qd treatment groups, respectively. After SAR439859 oral administration, the maximum impact on tumor [^18^F]-FES uptake inhibition was observed for the 12.5 mg/kg bid treatment group (*p* = 0.0012 versus vehicle group).

ERα IHC was scored for quantitative determination of the expression level of ERα in MCF7-Y537S tumor tissue for each animal (Fig. 2). ERα IHC characterization showed a high and homogenous expression of ER biomarkers in non-necrotic area. Following SAR439859 treatment at different doses, ERα expression decreased particularly in the zone near the necrosis compared to the control group; (3+) % nuclei were 89.6, 64.4, 51.3, and 61.9 in the vehicle, 25, 12.5, and 5 mg/kg groups, respectively. There was a mild positive correlation between [^18^F]-FES uptake and ERα ΙΗC H-score (Pearson correlation coefficient equal to 0.52).

### Therapeutic efficacy measured with [^18^F]-FDG imaging (experiment #3)

**Fig. 3:**
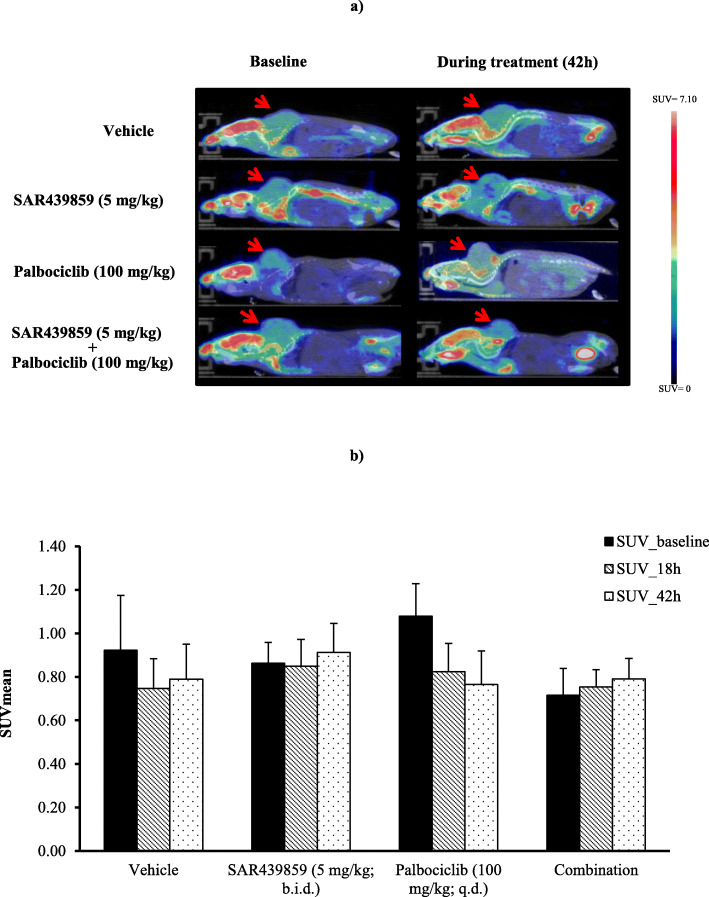
**a** Representative FDG-PET images of mice at baseline and during drug exposure. **b** Temporal PET-FDG quantification in MCF7-Y537S tumor upon treatment with SAR439859 alone or in combination with palbociclib for 3 days. Differences among groups were not statistically significant

**Fig. 4:**
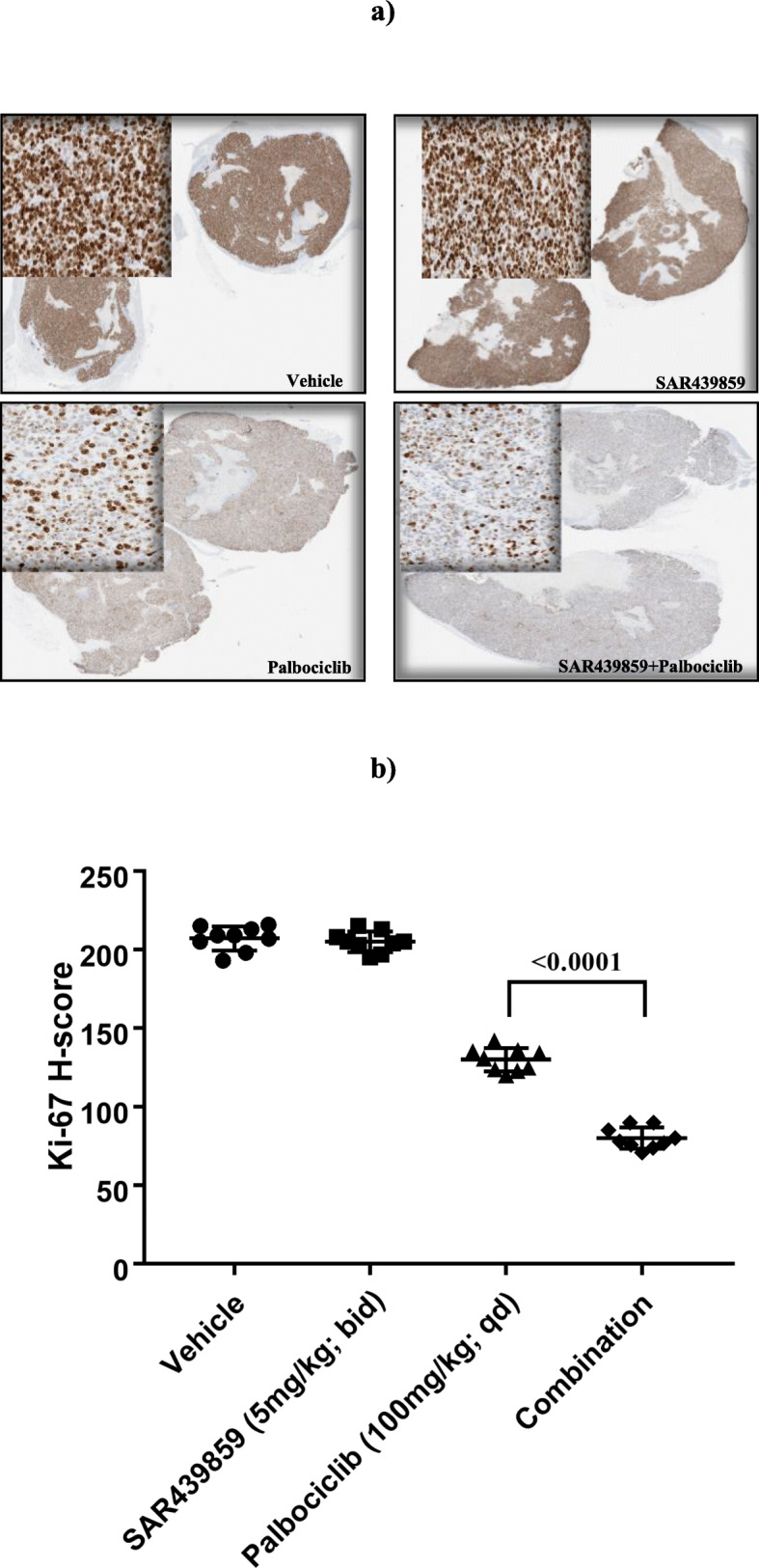
**a** Example of digital images of Ki-67 immunohistochemistry for each experimental group. **b** Analysis revealed that proliferation was severely impaired after palbociclib administration alone or in combination with SAR439859

Tumor metabolic activity as measured by FDG-PET imaging is represented for the 4 experimental groups in Fig. 3. No significant difference in SUVmean was seen in animals treated with SAR439859 (0.91 ± 0.13), palbociclib (0.76 ± 0.15), or the combination of the two compounds (0.79 ± 0.10) compared to baseline uptake at any time post-treatment. In this experiment, the proliferation status of each tumor was checked using standard Ki-67 labeling. Ki-67 immuno-labeling was significantly reduced (*p* < 0.0001) in all experimental groups (palbociclib: H-score = 130 ± 7.41; palbociclib+SAR439859: H-score = 80.1 ± 6.75) except that treated with SAR439859 only (H-score = 205.1 ± 6.63) compared to control (H-score = 206.9 ± 7.60) (Fig. 4). The combination treatment of SAR439859 (5 mg/kg/adm bid) and palbociclib (100 mg/kg/adm qd) demonstrated stronger anti-proliferative effect when compared to single drugs on the same dose regimen. The statistical analysis indicated that the combination was significantly different from palbociclib alone (*p* < 0.0001). This prompted us to evaluate the proliferation using non-invasive FLT-PET imaging.

### Therapeutic efficacy measured with [^18^F]-FLT imaging (experiment #4)

**Fig. 5:**
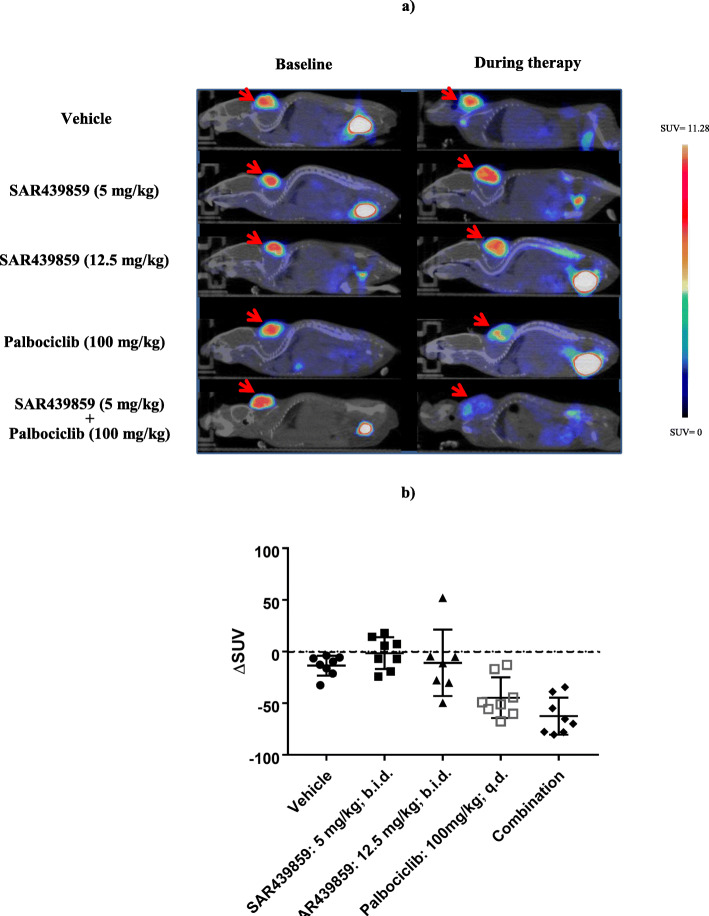
**a** Comparison of fused FLT-PET scans obtained before and during therapy induction. **b** Quantitative analysis of [^18^F]-FLT before and 72 h after treatment start with SAR439859 or palbociclib and the combination of the two. Data are expressed as ΔSUV (mean ± SD)

We investigated whether the combination therapy of SAR439859 and palbociclib could synergistically enhance anti-proliferative efficacy as measured by FLT-PET in the MCF7-Y537S xenograft model. We proposed to consider [^18^F]-FLT-PET to measure in vivo proliferation as a means of predicting the therapeutic response to SAR439859 and palbociclib or the combination of both agents. Figure 5 illustrates the mean tumor SUV of [^18^F]-FLT uptake; in comparison to the vehicle group, SAR439859 alone did not significantly modify the proliferation status of the tumor at any dose tested 72 h post-treatment (SUV_baseline_ = 5.85 ± 0.75 vs SUV_72h_ = 5.67 ± 0.51 at 5 mg/kg bid and SUV_baseline_ = 5.73 ± 1.27 vs SUV_72h_ = 4.77 ± 1.05 at 12.5 mg/kg bid). When combined to palbociclib, we observed a dramatic decrease (− 64%) in [^18^F]-FLT uptake compared to baseline. However, the reduction in uptake is not significantly different from the group dosed with palbociclib alone (− 46%) (*p* = 0.2412). Thus, we could not conclude from the FLT-PET data that the combination of the two compounds potentiates the anti-proliferative effect. Conversely, immunochemical analysis revealed a significant difference between the combination group (H-score = 57.37 ± 10.32) and palbociclib alone (H-score = 87.57 ± 15.99) in Ki-67 protein expression (*p* < 0.01). This latter result suggests that a synergic effect may occur between SERD and CDK 4/6 inhibitors.

## Discussion

In these preclinical experiments performed on an ERα-positive tumor model (MCF7-Y537S), we show that FES-PET can be used as a pharmacodynamic imaging biomarker to demonstrate ER target engagement and map impact of SAR439859 SERD. The study also shows that FLT-PET is useful to measure impact of palbociclib CDK 4/6 inhibitor on proliferation while suggesting that FDG-PET is inadequate to predict therapy efficacy in this experimental context.

Based on ER degradation kinetic profile (in vitro data not shown), we first assessed in vivo quantitative changes of SAR439859-induced ER occupation/degradation following a single administration and imaging at 4 h time-point (*in vitro*, the maximum effect appeared at 4 to 8 h in mice bearing MCF7-Y537S xenografts). We found a dose-dependent reduction of FES-PET signal with SAR439859 treatment with a maximum [^18^F]-FES uptake reduction of 50% at the highest dose tested of 12.5 mg/kg compared to the control group. A limitation of our study is that the global level of [^18^F]-FES uptake is weak in the MCF7-Y537S xenograft model; The mean SUV is between 0.30 and 0.42, compared to clinical cases where SUV is greater than 1.5–2.0 for ER-positive tumors [14]. Consequently, the dose-response effect might be hampered due to the limit of quantification of our PET scanner. However, in our experimental paradigm, the analysis of estrogen receptor expression levels by immunohistochemistry corroborated non-invasive FES-PET imaging to evaluate the effects of SAR439859. These results correlate with efficacy data where MCF7-Y537S tumor regression was observed when treated over 3 weeks at 12.5 mg/kg dose. The in vivo efficacy data also confirmed that the dose of 12.5 mg/kg administered twice daily can be considered the optimal dose since upper dosages did not significantly improve the anti-tumor activity [9].

In order to investigate [^18^F]-FDG and [^18^F]-FLT as pharmacodynamic biomarkers and support their potential use in phase 1 clinical trial, we explored the effectiveness of each radiotracer to measure the early functional response to SAR439859. FDG-PET imaging revealed no significant reduction in [^18^F]-FDG uptake for SAR439859 treatment groups at any dose tested. Kurland et al. [25] in a clinical study showed that the combination of FES-PET and FDG-PET could be effective in predicting the progression-free survival of ER+ patients, the follow-up with FDG-PET imaging only was not informative enough to guide therapy selection and dosing. In our study, the utility of FDG-PET imaging in measuring therapeutic efficacy under SAR439859 was not demonstrated; Heidari et al. [26] also concluded that the metabolic impact of fulvestrant was not detectable with [^18^F]-FDG after 3 days of treatment. Although FES-PET imaging provides early evidence that SAR439859 is effective in triggering ERα receptor modifications, a longer drug exposure period would be required to monitor downstream functional impacts (glycolytic or proliferative). In a different tumor model, He et al. [27] demonstrated lack of early significant outcomes with [^18^F]-FDG in animals administered with fulvestrant over 3 weeks. These preclinical data do not support the use of [^18^F]-FDG as an early sensitive pharmacodynamic biomarker for selective estrogen receptor degrader compounds such as SAR439859.

Our studies demonstrate that dual targeting of ERα and CDK 4/6 inhibitor can induce marked inhibition of [^18^F]-FLT uptake; this observation is associated with an enhanced inhibition of tumor growth compared to that observed with either single agent. The PET radiotracer [^18^F]-FLT thus represents a promising proof-of-concept surrogate biomarker for SERD therapy in combination with palbociclib.

The [^18^F]-FLT uptake decreases early after initiating the combination treatment with the CDK 4/6 inhibitor palbociclib and SAR439859; FLT-PET imaging appears as the most relevant imaging biomarker for early efficacy assessment in our experimental conditions. However, SAR439859 when injected alone did not show any impact upon [^18^F]-FLT uptake at any dose tested; the limited treatment duration is probably the reason for such observation. When measured with the caliper (data not shown), oral administration of SAR439859 induced significant tumor regression at 12.5 mg/kg (bid) starting 7 days after first administration, this indicates that longer exposure time to the drug is necessary to trigger downstream anti-proliferative effect on the MCF7-Y537S tumor model. The anti-proliferative effect measured in our study has been correlated with other biomarkers of proliferation namely Ki-67 immunohistochemistry. This robust proliferation index is significantly decreased in groups of animals treated with palbociclib alone or in combination. The difference in FLT-PET imaging between combination and palbociclib alone did not reach statistical significance; however, Ki-67 IHC revealed significant differences between those 2 groups, the intensity of staining decreasing more in the combination group. The reason of this discrepancy between FLT-PET imaging and IHC results might be due to the way data are collected; FLT-PET imaging data are expressed as SUVmean, i.e., the average uptake across the whole tumor, while for Ki-67, information is obtained from a limited number of tumor slices which might not be representative of the whole tumor and could bias the quality of quantification [28]. In their study, Pio et al. [29] concluded that FLT-PET imaging would be a valuable tool for predicting long-term clinical outcomes for women with breast cancer treated with endocrine therapy. Moreover, the authors noticed that [^18^F]-FLT changes were more closely correlated with the later changes in other pharmacodynamic biomarkers or morphometric analysis than were early [^18^F]-FDG changes.

Numerous preclinical and clinical data suggest that combination of cyclin inhibitors with SERD would improve the anti-tumoral efficacy especially in advanced breast cancer expressing ESR1 mutations [30]. Therefore, FLT-PET imaging appears as a valuable nuclear imaging modality for early visualization and monitoring of response to a combined targeted therapy [31]. Incorporating PET imaging into the development process of a new SERD therapeutic is highly relevant; recently Wang et al. [19] were able to determine the dosage of GDC-0810 to achieve 90% of ER+ receptors occupancy using FES-PET imaging; the authors concluded that patient dose selection could be carried out with FES-PET imaging. However, in this paper the authors did not present data of drug efficacy; a predictive biomarker such as FLT-PET imaging would have been of major interest for parallel assessment of treatment response.

As another illustration of proof-of-concept studies for investigation of compound efficacy with nuclear molecular imaging, it further suggests that preclinical imaging can provide important information on drug dosing and regimen which is relevant for clinical design [32]. The paradigm of implementing several PET tracers in order to monitor the tumor characteristics of each patient is of great promise; this remains a challenging objective, the achievement of which would improve the therapeutic management of patients [33, 34].

## Conclusion

In our preclinical experiments, target engagement measured with [^18^F]-FES-PET showed that the magnitude of uptake reduction is correlated with the administered dose of SAR439859. Moreover, [^18^F]-FES uptake correlated well with immunohistochemical scoring for ERα. As a biomarker of efficacy [^18^F]-FDG-PET was not adequate for early prediction and monitoring of therapy response since glucose metabolism may not be directly affected by endocrine therapy. In mice bearing subcutaneous MCF7-Y537S mutant ERα+ breast cancer model, [^18^F]-FLT effectively measures proliferation changes after the administration of the CDK 4/6 palbociclib inhibitor alone or in combination with SAR439859. In our study, [^18^F]-FES-PET is a valuable tool to non-invasively assess target engagement of SAR439859, allowing a quantitative assessment of ERα degradation.

## Data Availability

Please contact author for data requests.
